# CT Perfusion Metrics as Indicators of Intracranial Atherosclerotic Stenosis in Acute Ischemic Stroke: A Clinical Analysis

**DOI:** 10.2174/0115672026370562241223100210

**Published:** 2024-12-26

**Authors:** Yunpeng Liu, Jumei Huang, Jianwen Jia, Yingting Zuo, Yang Wang, He Liu

**Affiliations:** 1 Department of Neurosurgery, Beijing Chao-Yang Hospital, Capital Medical University, Beijing, China;; 2 Department of Neurology, Xuanwu Hospital, Capital Medical University, Beijing, China;; 3 Beijing Institute of Brain Disorders, Capital Medical University, Beijing, China

**Keywords:** Intracranial atherosclerotic stenosis, acute ischemic stroke, computed tomography perfusion, mechanical thrombectomy, endovascular therapy, predictive model

## Abstract

**Background:**

Intracranial Atherosclerotic Stenosis (ICAS) is a prevalent etiology of acute ischemic stroke (AIS), leading to significant morbidity and mortality. The accurate diagnosis and treatment of ICAS-induced AIS are critical to improving outcomes. This study assesses the application of Computed Tomography Perfusion (CTP) in predicting ICAS in AIS patients and its potential impact on patient management.

**Methods:**

A retrospective analysis was conducted on 224 AIS patients who underwent endovascular therapy (EVT) at one single Chinese Stroke Center between April 2022 and December 2023. Clinical and radiological data were collected, including patients’ demographics, CTP parameters, and 90-day modified Rankin Scale (mRS) scores. Logistic regression and receiver operating characteristic (ROC) curves evaluated the predictive power of CTP parameters for ICAS.

**Results:**

CTP analysis revealed significant differences in perfusion parameters between ICAS-induced AIS and other etiologies. ICAS patients had a smaller ischemic volume on admission and higher mismatch ratios [Time to Maximum, T_max_>6s: Other Causes: 132.4 [70.5, 183.3] mL, ICAS: 96.3 [79.8, 107.3] mL, *p* =0.0064; relative cerebral blood flow, rCBF<30%: Other Causes: 2.4 [0.0, 10.8] mL, ICAS: 0.6 [0.0, 7.0] mL, *p* =0.0145; mismatch ratio: 7.4 [2.5, 15.0], ICAS: 11.0 [4.6, 17.8], *p* =0.0285], indicating more salvageable brain tissue. The 90-day mRS showed better functional outcomes in the ICAS group, with a higher likelihood of minimal to no disability [mRS 90 equals 0-1: ICAS: 53.0% *vs*. Other Causes: 36.3%, *p* =0.0122]. The predictive model for ICAS, combining clinical manifestations and CTP parameters, yielded an area under the curve (AUC) of 0.7779, demonstrating good diagnostic performance.

**Conclusion:**

CTP is a valuable diagnostic tool for ICAS-induced AIS, offering the potential for early identification and informing the decision for endovascular treatment. The positive correlation between CTP findings and patient outcomes supports its utility in clinical practice.

## 1 INTRODUCTION

Acute Ischemic Stroke (AIS) represents a significant global health burden, being a leading cause of death and disability worldwide [1]. Primarily caused by a disturbance of blood supply to the brain, AIS usually results in substantial brain tissue damage and loss of brain function. While there are multiple causes for AIS, intracranial atherosclerotic stenosis (ICAS) is one of the key etiologies characterized by the narrowing of intracranial arteries due to atherosclerotic plaque buildup. The prevalence of ICAS as a cause of AIS varies geographically and ethnically, but it is recognized as a significant factor in stroke pathophysiology [2]. ICAS causes 30-50% of strokes in Asia and 8-10% of strokes in North America [3]. While other causes of AIS, like cardiac embolism, although they induce similar neurologic symptoms, have different therapeutic strategies. Not only the differences in pharmaceutical interventions, but thrombectomy, which often works for AIS caused by cardiac embolism, is usually not enough to build optimal recanalization and remedial treatments like ballooning and stenting of narrowed vessel segments are often considered for these cases. Thus, the clarification of the causes of AIS as early as possible helps the clinician to save time in making specific interventional strategies to save more penumbral brain tissue.

In the diagnostic landscape, computed tomography perfusion (CTP) has emerged as crucial tool in assessing the severity of ischemia at the time of patient admission because of its easy clinical accessibility. CTP utilizes a contrast agent to visualize cerebral hemodynamics over time, providing valuable insights into cerebral blood flow and volume, for the core infarction would gradually expand over time after stroke onset. This imaging modality is instrumental in differentiating between brain tissue that is permanently damaged and tissue that is potentially salvageable by reperfusion therapy [4]. The effectiveness of CTP in diagnosing AIS and determining thrombectomy eligibility has been highlighted in recent studies, underscoring its role in acute stroke management [5, 6]. In ICAS-AIS (ICAS induced AIS), where the pathophysiology may involve complex patterns of blood flow obstruction and potential collateral circulation, CTP provides an invaluable window into the cerebral vascular status. The quantification of perfusion parameters in CTP can potentially unveil subtle hemodynamic changes characteristic of ICAS, thereby assisting in distinguishing it from other stroke etiologies. The application of CTP in ICAS-AIS thus holds significant promise in enhancing diagnostic accuracy.

In our current research, we delved deeper into the comparative analysis of CTP characteristics, mainly relative cerebral blood flow (rCBF) and Time to Maximum (T_max_), between ICAS-induced AIS and AIS resulting from other etiologies. This comparative approach has unveiled distinct differences in several key perfusion parameters. Notably, we observed variations in cerebral blood flow and volume measurements, as well as in the time to peak perfusion. Moreover, a statistic model was built based on our study to identify ICAS-AIS, which reached an ideal predictive value. These findings provide insights into the varying degrees of ischemic severity and show the potential for brain tissue salvageability across different causes of AIS.

## 2 METHODS

### 2.1 Patients

This retrospective study involved a total of 224 patients with AIS who underwent endovascular therapy at our hospital, from April 1^st^, 2022, to December 31^st^, 2023. All patients provided informed consent for their inclusion in the study. The research protocol was reviewed and approved by the Ethics Committee of Capital Medical University [Approval No. 2022-KE-268]. The study was conducted in accordance with the principles outlined in the Declaration of Helsinki, ensuring ethical standards were upheld and patient confidentiality was maintained.

### 2.2 Clinical Data Collection

We systematically collected a comprehensive set of clinical data for each patient, including demographic information (age and gender), time from symptom onset to clinic, blood pressure and blood glucose levels, neurological status as assessed by the National Institutes of Health Stroke Scale (NIHSS) upon admission, and the door-to-puncture time (DPT). We also recorded the presence of complications or comorbidities such as hypertension, diabetes, atrial fibrillation, coronary artery disease, and smoking history. Additionally, information was gathered on whether patients received intravenous thrombolysis prior to EVT. The prognosis was evaluated by the patients’ 90-day modified Rankin Scale (mRS 90) scores after the onset of the disease.

### 2.3 Etiology Identification

Our experienced physicians and radiologists made a clear distinction between ICAS and other causes of AIS, adhering to precise criteria by the European Stroke Organization [7]. Specifically, the diagnosis of an ICAS-AIS was contingent upon [1] the presence of significant stenosis or blockage, exceeding 50%, in either an extracranial or intracranial artery on the same side; [2] no high-density sign along the cerebral arteries found in non-enhanced CT; [3] remained stenosis after three times of thrombectomy; [4] early re-occlusion directly after thrombectomy.

### 2.4 CTP Parameter Collection

All radiologic information was collected by multimodal CT before EVT (including non-enhanced CT, CT angiography, and CTP) using a CT machine (GE, USA). Imaging parameters were standardized with a field of view (FOV) set at 51.2 x 51.2 cm, dimensions (Dims) at 512 x 512 pixels, and a zoom factor of 1.41x to maintain resolution integrity. The window width/level (w/l) settings were optimized at 1024/512 for ideal contrast resolution in stroke detection. The initial CTP images underwent automated processing with SHUKUN AI perfusion software (SK-CTPDoc, StrokePro V2.0, SHUKUN Technology, Beijing, China), resulting in the generation of 4 distinct parameter maps: CBF, CBV, MTT, and T_max_. This process swiftly identified the infarct core, defined by a rCBF below 30%, and the volume of the ischemic region, indicated by a T_max_ exceeding 6 seconds. Additionally, the software automatically calculated the mismatch ratio. The software selected the artery input and venous outflow thresholds automatically for perfusion analysis. However, manual adjustments were made to the selection of arterial input and venous outflow upon observing an abnormal time–density curve (TDC), which indicated incorrect artery and vein selection. The mismatch ratio was calculated as the volume of T_max_>6 seconds divided by the volume of rCBF<30%.

### 2.5 Predictive Model

We used logistic regression modelling to construct a prediction model for diagnosis of ICAS. The predictive ability of the models was estimated by internal validation with the area under the receiver operating characteristic (ROC) curve and diagnostic efficiency parameters (C statistic). The nomogram prediction model was constructed based on the results of the regression analyses.

### 2.6 Statistical Methods

All statistical analyses were conducted using GraphPad Prism version 10.0 (GraphPad Software, MA, US). The regression analysis, nomogram prediction model, and ROC curve analysis were performed using R (version 4.3.0). The Chi-Square test was employed for analyzing nonparametric data, while the student’s t-test was used for parametric data, represented as Mean±Standard Deviation. The Mann-Whitney test was used for non-parametric data, represented as Median (25^th^ quartile, 75^th^ quartile). A *P*-value of less than 0.05 was considered statistically significant in all analyses.

## 3 RESULTS

### 3.1 Clinical Characteristics of Subjects

From April 2022 to September 2023, our hospital managed a total of 224 patients suffering from AIS with large vessel occlusion. These patients were categorized based on the underlying causes: 100 patients were identified with ICAS, while the remaining 124 patients had strokes due to other causes; the differentiation of ICAS and other causes of AIS-LVO (acute ischemic stroke with large vessel occlusion) was illustrated as Fig. (**1**). A notable observation was the significant difference in the average age of patients in these groups. Patients with ICAS had a younger average age of 64.5±11.5 years, compared to 68.8±10.4 years for those with other causes (*P* =0.0037). Additionally, it was noted that there was no significant difference in gender distribution across both groups. Regarding comorbid conditions, a significantly lower incidence of atrial fibrillation was observed in the ICAS group (Other Causes: 33.9%, ICAS: 6%, *P* <0.0001). However, the prevalence of other comorbidities, such as hypertension, diabetes, coronary artery disease, and smoking, showed no significant differences between the two groups. Furthermore, there were no significant differences in the systolic blood pressure and serum glucose levels upon admission between the two patient groups.

In terms of clinical presentation, the National Institutes of Health Stroke Scale (NIHSS) scores at admission were significantly lower for the ICAS group compared to those with other causes. Additionally, the Door to Puncture Time (DPT), a critical measure in stroke management, was comparable between both groups, indicating a uniform efficiency in the initial management and intervention processes for all AIS-LVO patients in our center during this period (Table **1**).

### 3.2 AIS Patients with ICAS have Smaller Ischemic Volume on CTP on Admission

In the assessment of computed tomography perfusion (CTP) scans on admission, the representative image of CTP in patients with ICAS and with other causes are illustrated in Fig. (**2**).

**Fig. (3):**
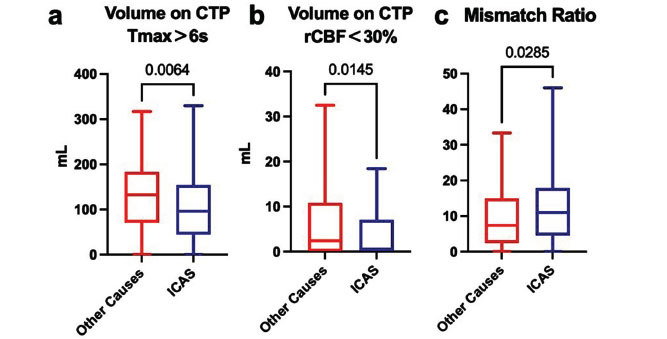
Comparison of CTP parameters of AIS patients with ICAS and other causes. (**a**) The volume of brain tissue with T_max_ > 6 seconds was significantly smaller in the ICAS group (96.3 mL) compared to other causes (132.4 mL, *P* = 0.0064). (**b**) The volume of brain tissue with rCBF < 30% was also significantly reduced in ICAS patients (0.6 mL *vs*. 2.4 mL, *P* = 0.0145). (**c**) ICAS patients exhibited a significantly higher mismatch ratio (11.0 *vs*. 7.4, *P* = 0.0285), reflecting a greater proportion of salvageable brain tissue.

Patients with AIS-LVO attributed to ICAS demonstrated significantly smaller volumes of brain tissue with T_max_>6s (Other Causes: 132.4 (70.5, 183.3) mL, ICAS: 96.3 (79.8, 107.3) mL, *P* =0.0064, Fig. **3a**). Furthermore, the volume of brain tissue exhibiting rCBF<30% was also significantly reduced in patients with ICAS (Other Causes: 2.4 (0.0, 10.8) mL, ICAS: 0.6 (0.0, 7.0) mL, *P* =0.0145, Fig. **3b**).

Following the initial findings, further analysis of CTP parameters revealed a significant difference in the mismatch ratio between groups. Patients with AIS due to ICAS exhibited a higher mismatch ratio compared to those with other etiologies (Other Causes: 7.4 (2.5, 15.0), ICAS: 11.0 (4.6, 17.8), *P* =0.0285, Fig. **3c**), indicating a greater amount of salvageable brain tissue.

### 3.3 Evaluation of 90-day Outcomes in AIS Patients with ICAS *versus* Other Causes

Following the onset of the current ischemic stroke event, outcomes were evaluated using the modified Rankin Scale (mRS) at 90 days (mRS 90). Analysis revealed that patients with ICAS exhibited a significantly higher likelihood of achieving better functional outcomes compared to those with other etiologies. Specifically, a greater proportion of ICAS patients achieved a score of 0-1 on the mRS, indicating minimal to no disability (ICAS: 53.0% *vs*. Other Causes: 36.3%, *P* =0.0122). Additionally, the percentage of patients scoring 0-2 on the mRS, reflective of slight disability, was also notably higher in the ICAS group (ICAS: 58.0% *vs*. Other Causes: 38.7%, *P* =0.0031). Furthermore, the proportion of individuals scoring 0-3 on the mRS, corresponding to moderate disability but still able to walk unassisted, was significantly greater among ICAS patients (ICAS: 66.0% *vs*. Other Causes: 52.8%, *P* =0.0406). These findings, visualized in Fig. (**4**), underscore the distinct recovery trajectories of patients with ICAS when subjected to EVT.

### 3.4 Predictive Model of ICAS in Patients with AIS

The primary logistic regression showed that Atrial Fibrilization (β=-1.17, 95% CI= -2.12 to -0.22, *P* =0.02) was independently correlated to ICAS diagnoses (Table **2**). We compared the model using clinical characteristics with and without CTP parameters, including rCBF<30% and T_max_>6s (Table **3**). The C statistic for ICAS diagnoses was 0.77 without CTP parameters, including the main effect and interaction effect, and was also 0.78 by adding a mismatch rate. The C statistic did not significantly improve with the addition of CTP data (C statistic difference=0.006; 95% CI= -0.01 to 0.01, *P* =0.55). The C statistic for mRS score 0-1 by using the combined model was 0.88 (0.83-0.92), significantly higher (*P* =0.04) compared to the conventional model of 0.85 (0.80-0.90). This prediction model shows better efficacy in the prediction of good functional outcomes.

**Fig. (5):**
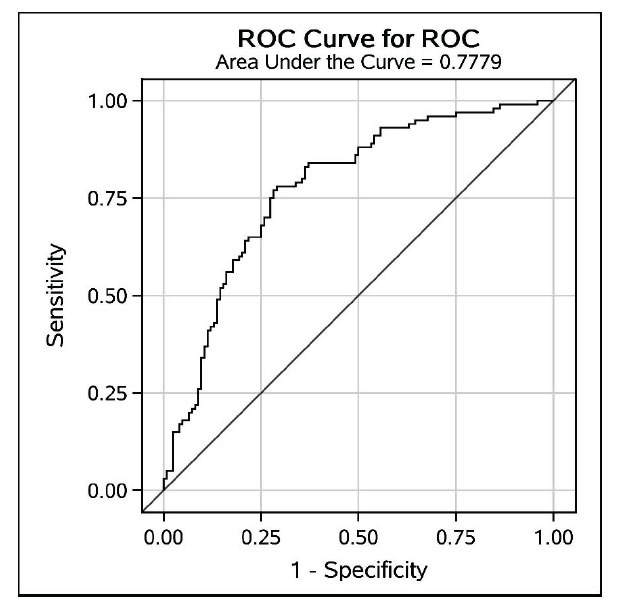
ROC Curve for ICAS (Assessment combining conventional data and rCBF + T_max_). The ROC curve illustrates the predictive performance of the final logistic regression model for identifying ICAS-related AIS. The model, incorporating age, sex, NIHSS on admission, smoking status, time from onset to admission, and CTP parameters (rCBF < 30% and T_max_ > 6s), achieved an area under the curve (AUC) of 0.7779, indicating good diagnostic accuracy.

**Fig. (6):**
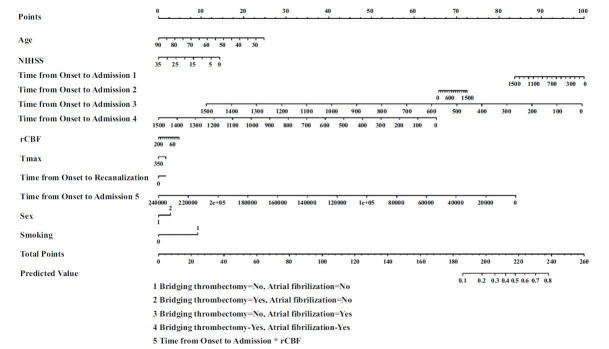
Nomogram for ICAS prediction. The nomogram represents the final logistic regression model for predicting the likelihood of ICAS in AIS patients. Each variable is assigned a point value, and the total points predict the probability of ICAS, providing a practical tool for clinical decision-making.

The final logistic regression included age, sex, NIHSS on admission, smoking, time from onset to admission, volumes of rCBF<30%, and T_max_>6s. The AUC of this model was 0.7779 (Fig. **5**). A nomogram was composed based on the final model to provide references to clinical practice (Fig. **6**).

## 4 DISCUSSION

AIS continues to be a leading cause of morbidity and mortality globally, posing a significant public health challenge. ICAS, characterized by the narrowing of intracranial arteries due to atherosclerotic plaque buildup, plays a critical role in the pathophysiology of AIS [8]. The geographical and ethnic variations in the prevalence of ICAS underscore its complex nature and the need for targeted diagnostic and therapeutic strategies [9]. Recent advancements in imaging technologies, particularly CTP, have revolutionized the acute management of ischemic stroke by enabling the precise assessment of the hemodynamics of brain tissue [10-12]. Our study contributes to this evolving landscape by providing novel insights into the CTP characteristics of AIS induced by ICAS compared to other etiologies. By elucidating the distinct perfusion patterns associated with ICAS, our research underscores the potential of CTP in enhancing diagnostic accuracy, informing treatment decisions, and ultimately improving patient outcomes in this specific subset of stroke patients.

In the context of AIS due to ICAS, our findings align with broader clinical observations that patients with lower NIHSS scores at admission represent a distinct subgroup within the stroke population. Notably, a study by Kniep *et al*. corroborates the observation that lower NIHSS scores at admission are strongly associated with better clinical outcomes following thrombectomy for middle cerebral artery occlusions, a finding that resonates with the characteristics of ICAS-induced AIS observed in our cohort [13]. The study by Abbas *et al*. also supports that thrombectomy is effective and safe for patients with large vessel occlusions presenting with low NIHSS scores [14]. The observed better prognosis in patients with lower NIHSS scores may reflect a more robust cerebrovascular reserve and a greater capacity for neuroplasticity, facilitating recovery after AIS. This subgroup might also have fewer comorbidities, contributing to their resilience against ischemic injury and enhancing their response to reperfusion therapies like EVT. Additionally, the distinct pathophysiology of ICAS, involving localized arterial stenosis, might not lead to as severe neurological deficits initially.

The observed differences in CTP parameters for ICAS patients-namely, lower volumes of T_max_>6s and rCBF <30%, along with higher mismatch ratios compared to other stroke causes-may also reflect ICAS's unique pathophysiology. Lower T_max_>6s and rCBF<30% volumes suggest that ICAS-induced strokes might involve smaller core infarct areas, possibly due to more effective collateral blood flow mitigating the extent of critically underperfused tissue. The higher mismatch ratio indicates a larger portion of brain tissue that, despite being at risk, remains salvageable, highlighting the potential for reperfusion treatments to be particularly beneficial in ICAS patients. In the study by Chen *et al*., the prognostic significance of CTP parameters in predicting outcomes for thrombolysis-treated AIS patients was rigorously analyzed through univariable and multivariable regression models. The researchers found that among the predictors analyzed, the infarct core volume determined by CTP emerged as the only independent factor significantly associated with functional outcomes, and a larger infarct core volume determined by CBF was associated with an increased risk of poor outcomes [15]. Our study found that, combined with a conventional predictive model of clinical representations, CTP parameters showed efficacy in predicting better neurological outcomes for patients with AIS. Another reason for this might be that a larger core infarct volume usually correlates with occlusion of a more proximal artery, like a carotid artery, compared to the middle cerebral artery or M1 segment compared to the M2 segment, thus more severe neurological deficits [16].

The notably better functional outcomes observed in ICAS patients following AIS, as evidenced by their superior performance on the 90-day mRS, could be significantly attributed to the efficacy of EVT. In contrast to ICAS, patients with strokes of other etiologies, such as cardioembolic strokes, may not experience the same level of benefit. These emboli, either from the heart or other organs, could lead to more diffuse or multifocal areas of cerebral ischemia, which may not be as amenable to direct mechanical revascularization as the localized stenosis seen in ICAS [17]. Furthermore, cardioembolic strokes carry a higher risk of recurrent embolization. They may be associated with larger infarct sizes by the time of intervention, both of which can contribute to worse functional outcomes despite successful thrombectomy, compared to ICAS.

Recent advancements in high-resolution vessel wall magnetic resonance imaging (HR-MRI) have significantly enhanced the ability to distinguish ICAS from other causes of ischemic stroke. Chen *et al*. emphasized that HR-MRI detects not only luminal stenosis but also provides critical insights into plaque morphology, including intraplaque hemorrhage, fibrous cap integrity, and gadolinium enhancement, which are pivotal in identifying symptomatic ICAS plaques compared to inflammatory vasculopathy or dissection [18]. Sanchez *et al*. further highlighted that HR-MRI outperforms conventional luminal modalities like CTA and MRA by characterizing non-stenotic, outwardly remodeled plaques and detecting perfusion deficits due to distal hemodynamic compromise. These imaging capabilities enable a more accurate classification of ICAS as a distinct stroke mechanism [19]. Additionally, HR-MRI findings can help differentiate artery-to-artery embolism and branch occlusion by mapping plaque burden and irregularities in specific intracranial segments [20]. Future studies integrating HR-MRI with advanced computational analyses, combined with complementary tools such as CTP, may offer deeper insights into the hemodynamic interactions and the structural characteristics of thrombosis associated with ICAS plaques.

This study has several limitations that warrant consideration. First, the relatively small sample size and single-center design may limit the generalizability of the findings. Future research should focus on multicenter studies with larger, more diverse populations to validate these results across different demographic and clinical settings. Second, while this study primarily utilized CTP for diagnostic evaluation, incorporating post-thrombectomy MRI could provide complementary insights into vascular remodeling and infarct evolution in ICAS. Third, this study did not evaluate the impact of variations in endovascular treatment protocols on CTP metrics and outcomes. Prospective studies assessing the interplay between individualized EVT strategies and CTP findings are needed to refine treatment paradigms. Lastly, the use of advanced AI-driven imaging analysis tools to integrate CTP with other diagnostic modalities, such as DSA or transcranial Doppler ultrasound, may enhance the precision of ICAS diagnosis and prognosis. Addressing these limitations in future research will contribute to a more comprehensive understanding of ICAS-related AIS and its optimal management strategies.

## CONCLUSION

To sum up, our study underscores the utility of CTP in identifying ICAS as a cause of AIS and in aiding tailored endovascular interventions. The research sheds light on unique CTP patterns indicative of ICAS. The findings imply that ICAS patients typically have smaller core infarcts and more brain tissue amenable to recovery, suggesting better outcomes with appropriate treatment. Further studies with larger samples and more diverse imaging modalities are advised to refine our predictive model and improve stroke care strategies.

## Figures and Tables

**Fig. (1):**
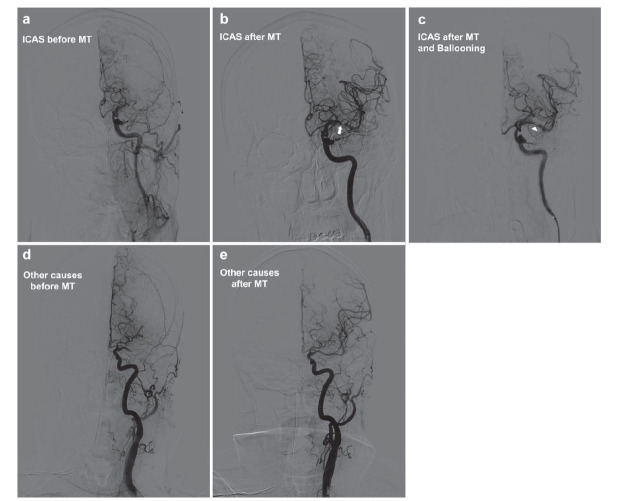
Illustration of etiology differentiation of AIS-LVO according to DSA image before and after MT. (**a**-**c**) A patient with acute occlusion of the left middle cerebral artery showing severe stenosis remained after MT (white arrow), and the stenosis even remained after rescue ballooning (white arrowhead). This patient was diagnosed with ICAS. (**d** and **e**) A patient with non-ICAS occlusion of the left middle cerebral artery, in this case, the cardiac embolism, was diagnosed.

**Fig. (2):**
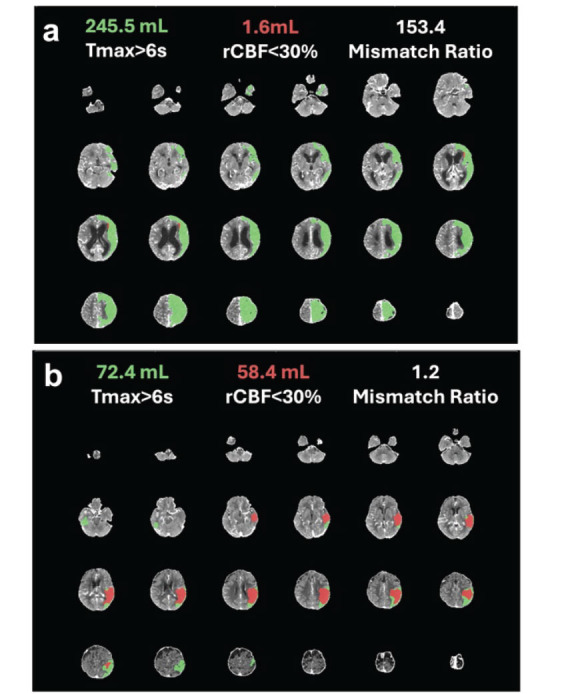
In CTP scans between AIS patients of ICAS and Other Causes, the green color shows the brain area of T_max_>6s, and the red color shows the area of rCBF<30%. (**a**) CTP of a patient with ICAS. (**b**) A patient with other causes of AIS, in this case, cardiac embolism.

**Fig. (4):**
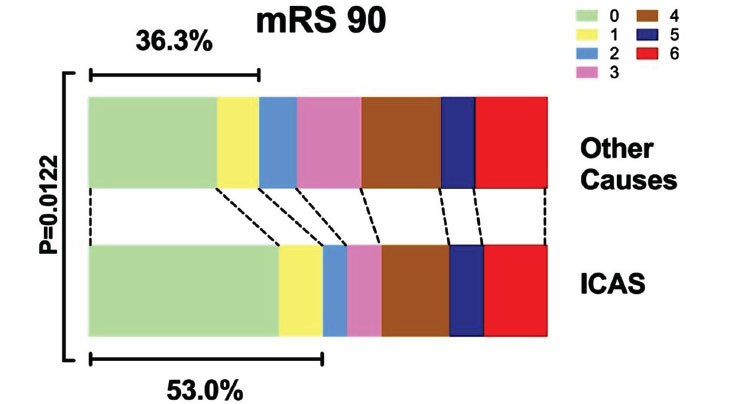
Modified Rankin Scale (mRS) Scores at 90 Days for ICAS and Other AIS Etiologies. A significantly higher proportion of ICAS patients achieved mRS 0-1 (53.0% *vs*. 36.3%, *P* = 0.0122) and mRS 0-2 (58.0% *vs*. 38.7%, *P* = 0.0031) compared to non-ICAS patients, indicating better recovery. These results suggest more favorable outcomes in ICAS patients, underscoring the importance of tailored EVT strategies for this subgroup.

**Table 1:** AIS patient characteristics of ICAS and other causes.

**-**	**Other Causes (n=124)**	**ICAS (n=100)**	***P* Value**
**Age**	68.8 ± 10.4	64.5 ± 11.5	*P *=0.0037
**Gender (Male)**	84 (67.7%)	77 (77.0%)	*P* =0.1255
**Co-Morbidity**			
**Atrial Fibrilization**	42(33.9%)	6 (6.0%)	*P *<0.0001
**Hypertension**	85 (68.6%)	67 (67.0%)	*P *=0.8052
**Diabetes**	37 (29.8%)	27 (27.0%)	*P *=0.6401
**Coronary Artery Disease**	24 (19.7%)	14 (14.0%)	*P *=0.2743
**Smoking**	42 (33.9%)	41 (41.0%)	*P *=0.2721
**Systolic Blood Pressure on Admission (mmHg)**	142.0 (130.0, 171.8)	147.0 (130.5, 166.0)	*P *=0.8518
**Serum Glucose Level on Admission (mmol/L)**	7.1 (6.2, 8.4)	7.3 (6.3, 10.2)	*P *=0.2616
**NIHSS on Admission**	8 (0, 15)	3 (1, 12)	*P *=0.0362
**Bridging Thrombectomy**	10 (10.0%)	24 (16.9%)	*P *=0.1282
**DPT**	89.0 (79.0, 113.0)	91 (79.8, 107.3)	*P *=0.9696

**Table 2:** Observed main effects in cohort (with mismatch ratio).

**-**	**β (95% CI)**	***P* Value**
**Age per year**	-0.03 (-0.05 to -0.0003)	0.08
**Gender**	-	-
**Female**	Reference	0.61
**Male**	-0.10 (-0.46 to 0.27)	-
**NIHSS on admission per point**	-0.03 (-0.07 to 0.02)	0.21
**Bridging thrombectomy**	-	-
**No**	Reference	0.97
**Yes**	-0.02 (-0.84 to 0.81)	-
**Atrial fibrilization**	-	-
**No**	Reference	0.02
**Yes**	-1.17 (-2.12 to -0.22)	-
**Smoking**	-	-
**No**	Reference	0.09
**Yes**	0.31 (-0.05 to 0.68)	-
**Volume of rCBF<30% (mL)**	0.001 (-0.04 to 0.03)	0.92
**Volume of T_max_>6s (mL)**	-0.002 (-0.007 to 0.002)	0.29
**Time from onset to admission per min**	-0.002 (-0.007 to 0.003)	0.45
**Time from onset to recanalization per min**	0.001 (-0.002 to 0.005)	0.49
Volume of rCBF<30% * Time of onset	-0.00002 (-0.0001 to 0.00006)	0.58
Bridging thrombectomy * Time onset	-0.002 (-0.004 to 0.0007)	0.17
Atrial fibrilization * Time of onset	0.0005 (-0.002 to 0.003)	0.64

**Table 3:** Reclassification and discrimination statistics for outcomes by mismatch ratio.

**-**	**C-statistic**		
	**Estimate (95% CI)**	**Difference (95% CI)**	***P* value**
**ICAS**	-	-	-
**Conventional model**	0.77 (0.71-0.83)	-	-
**Conventional model + rCBF and T_max_**	0.78 (0.72-0.84)	0.006 (-0.01 to 0.02)	0.55
**mRS 0-1**	-	-	-
**Conventional model**	0.85 (0.80-0.90)	-	-
**Conventional model + rCBF and T_max_**	0.88 (0.83-0.92)	0.03 (0.001 to 0.05)	0.04

## Data Availability

All data generated or analyzed during this study are included in this published article.

## LIST OF ABBREVIATIONS

AIS: Acute Ischemic Stroke
AUC: Area Under the Curve
CTP: Computed Tomography Perfusion
DPT: Door-to-puncture Time
EVT: Endovascular Therapy
FOV: Field of View
ICAS: Intracranial Atherosclerotic Stenosis
NIHSS: National Institutes of Health Stroke Scale
ROC: Receiver Operating Characteristic

## STANDARDS OF REPORTING

STROBE guidelines were followed.
